# Causal relationship between physical activity and platelet traits: a Mendelian randomization study

**DOI:** 10.3389/fphys.2024.1371638

**Published:** 2024-03-20

**Authors:** Mingyuan Jia, Zhiyong Wang, Fengting Hu

**Affiliations:** Department of Physical Education, Dong-A University, Busan, Republic of Korea

**Keywords:** physical activity, platelet traits, Mendelian randomization, causal relation, physiological indices

## Abstract

**Introduction:** The purpose of this study was to discuss the causal relationship between physical activity and platelet traits.

**Methods:** A dataset from a large-scale European physical activity and platelet traits was collected by using Mendelian randomization of the study. For the analysis, the inverse variance weighting method, weighted median and MR-Egger were used to estimate causal effects. The sensitivity analyses were also performed using Cochran’s Q test, funnel plots and Leave-one-out analysis.

**Results:** Light DIY, other exercises, strenuous sports, walking for pleasure were significantly associated with a decrease in platelet crit. But none of the heavy /light DIY was associated with increase in platelet crit. Other exercises and strenuous sports were associated with decrease in platelet count.

**Conclusion:** Some types of physical activity have a causal relationship with platelet crit and platelet count. However, the types of physical activity we studied have not supported a causal relationship with mean platelet volume and platelet distribution width.

## Introduction

Platelets are produced by the bone marrow and play a crucial role in maintaining vascular integrity and preventing physiological and pathological processes such as hemorrhage (Salles et al., 2008), inflammation, tumor metastasis (Schiffer et al., 2018; Pogorzelska et al., 2020), in which functional characteristics are emerging and have important genetic implications (Bunimov et al., 2013). However, some of the traits of platelets may be altered due to several diseases, thereby increasing the likelihood of developing platelet-related disorders. By way of example, platelet counts are significantly lower in critically ill COVID-19 patients (Jiang et al., 2020; Lippi et al., 2020), and 58%–95% of critically ill patients suffer from mild thrombocytopenia (Fu et al., 2020; Levi et al., 2020; Thachil, 2020). Moreover, many diseases can affect platelet traits. The platelet count level is higher, and the mean platelet volume (MPV) is lower in patients with active disease (Zareifar et al., 2014). Reactive thrombocytosis and higher platelet specific volume and platelet distribution width (PDW) are frequent in tuberculosis disease, with a relationship between thrombocytosis and inflammatory response (Sahin et al., 2012). Therefore, improving platelet function and treating platelet-related disorders might be beneficial if platelet traits or platelet indices can be improved.

Many studies have been conducted to point out that physical activity, exercise, or other types of sports can have different effects on platelet counts and MPV. The average platelet volume decreases in hematological parameters measured after exercise (Boyali et al., 2018; Durmuş et al., 2021). Some studies have also shown increased MPV after exercise (Bachero-Mena et al., 2017; erdoğan, 2020). However, some findings have also demonstrated no change in MPV after exercise (Lundberg Slingsby et al., 2017; Tóth-Zsámboki et al., 2017; Heber et al., 2020). Likewise, there are different conclusions about changes in platelet counts after exercise, however more studies have obtained the result that platelet counts do not change after exercise training (Coppola et al., 2004; Wang and Chow, 2004; Bachero-Mena et al., 2017; Lundberg Slingsby et al., 2017; Boyali et al., 2018; erdoğan, 2020; Heber et al., 2020) and relatively few studies have obtained a decrease in platelet counts (Burri et al., 1996) and an increase in platelet counts (Tóth-Zsámboki et al., 2017). Findings from another study on PDW, one of the platelet traits, showed different results in different populations with varying exercise modalities or other sports programs. The difference in PDW between athletes and sedentary college students was not significant (*p* > 0.05) (Kırbaş et al., 2015). Healthy people also showed no substantial changes in PDW after long-distance runs such as marathons (Kratz et al., 2006). However, results from another study that analyzed hematological parameters in male handball players before and after a match showed decreased PDW after the (Koc et al., 2012). There are fewer studies on the effect of physical activity or exercise, compared to the first three indices, etc., on the platelet-specific volume. The studies that have been published are mostly cross-sectional and have shown that there is still ambivalence regarding the effect of exercise modalities such as physical activity on platelet traits, and the causal relationship between physical activity and platelet traits remains unclear.

Since the possibility of reverse causality and confounding cannot be completely ruled out using observational studies because of the inherent shortcomings of traditional designs, the chance of biased associations and conclusions is highly probable (Sekula et al., 2016). While Mendelian randomization (MR) can assess possible causality between modifiable exposures and outcomes using the genetic variants associated with them and minimize the potential for bias due to confounding and reverse-direction causality (Zhuang et al., 2020; Skrivankova et al., 2021). Given the uncertainty of the causal relationship between physical activity and platelet shape, for this study, we have assessed the potential causal effect of physical activity on four platelet traits (MPV, platelet crit, PDW, and platelet count) using an MR design with data from a Large Genome-Wide Association Study (GWAS).

## Methods

### Study design

**FIGURE 1:**
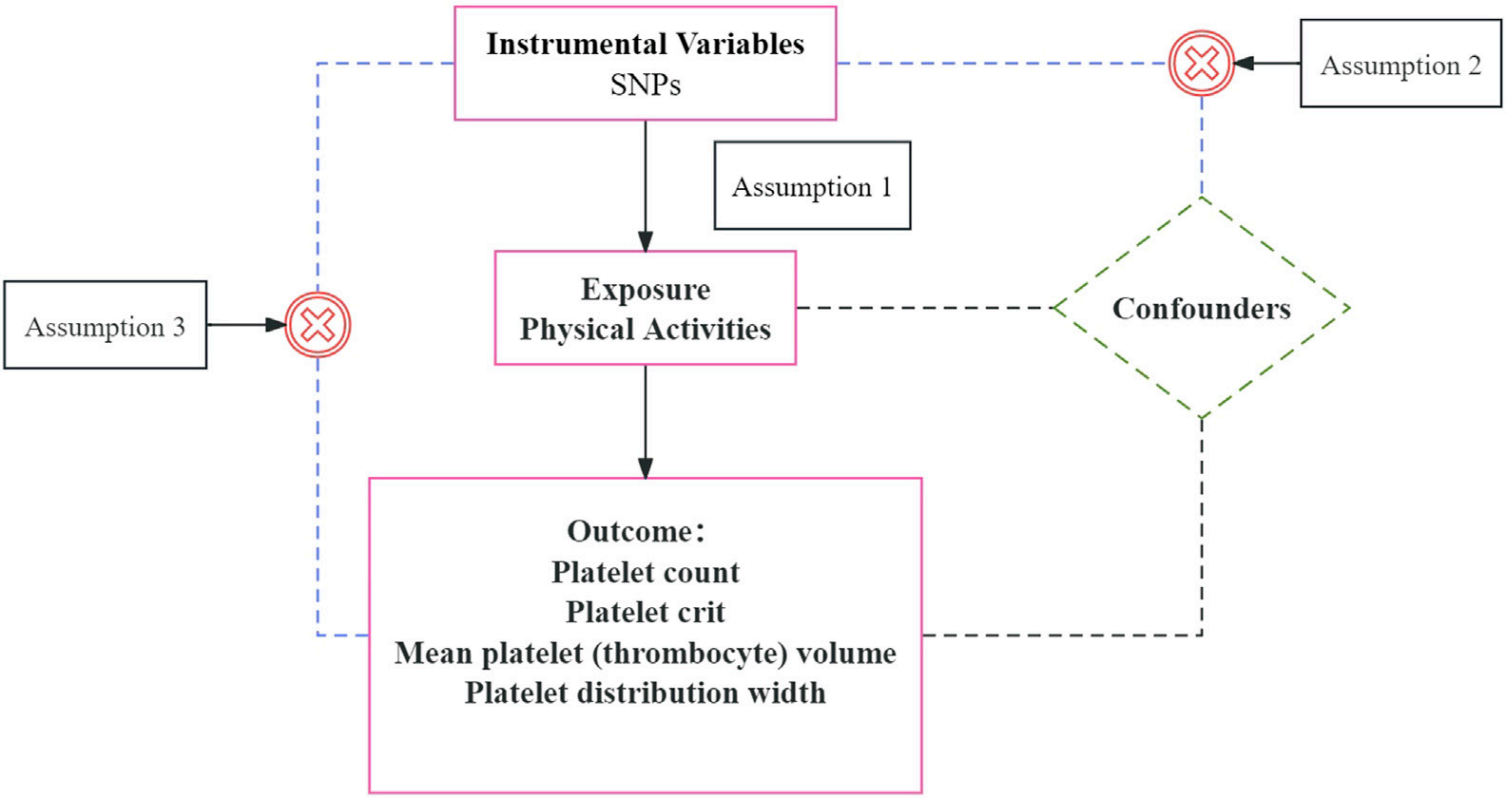
Mendelian randomization hypothesis.

Our two-sample MR study relied on summary-level data from the published and available OpenGWAS. No additional ethical approvals were required because the data were reanalyzed at the abstract level. MR was analyzed using genetic instrumental variables (IVs) to assess the causal relationship between exposure and outcome. The MR analysis was based on the following three core assumptions (as shown in Figure 1): 1) The selected IVs must be significantly associated with exposure (PA) (Lawlor et al., 2008; Sekula et al., 2016). 2) It is independent of the complexity of the exposure-outcome association (Pierce et al., 2011). 3) IV affects outcome only through exposure (PA) and has no other pathway to affect outcome (Pierce et al., 2011). The MR should be able to infer causality without unmeasured confounding and reverse causality when these assumptions are being fulfilled (Pierce et al., 2011).

### Data source

GWAS summary statistics for exposure were obtained from the OpenGWAS database, which included 460,376 individuals of European ancestry (222,470 cases and 237,906 controls) (Table 1). GWAS data for platelet traits were obtained from a GWAS conducted on UK Biobank participants, which included 350,474 participants.

**TABLE 1:** Data set information.

Trait	Year	Population	Sex	Cases	Controls	Sample size	Data source
TPA: Heavy DIY	2018	European	M&F	197,006	263,370	460,376	https://gwas.mrcieu.ac.uk/
TPA: Light DIY	2018	European	M&F	236,244	224,132	460,376	https://gwas.mrcieu.ac.uk/
TPA: None of the Heavy DIY or Light DIY	2018	European	M&F	28,040	432,336	460,376	https://gwas.mrcieu.ac.uk/
TPA: Other exercises	2018	European	M&F	222,470	237,906	460,376	https://gwas.mrcieu.ac.uk/
TPA: Strenuous sports	2018	European	M&F	47,468	412,908	460,376	https://gwas.mrcieu.ac.uk/
TPA: Walking for pleasure	2018	European	M&F	329,755	130,621	460,376	https://gwas.mrcieu.ac.uk/
Platelet count	2018	European	M&F			350,474	https://gwas.mrcieu.ac.uk/
Platelet crit	2018	European	M&F			350,474	https://gwas.mrcieu.ac.uk/
Mean platelet (thrombocyte) volume	2018	European	M&F			350,474	https://gwas.mrcieu.ac.uk/
Platelet distribution width	2018	European	M&F			350,474	https://gwas.mrcieu.ac.uk/

### Instrument variables selection

In this MR study, single nucleotide diversity (SNPs) of genome-wide significance were extracted from the GWAS database as IV. The correlation between instrumental variables and physical activity was assessed using broader thresholds (*p* < 1 × 10^−5^). The specific process of screening was as follows: 1) SNPs capable of being analyzed were extracted from the pooled data of different physical activity types over the past 4 weeks using the TwoSampleMR extension package of the R software. 2) SNPs with LDr^2^<0.01, kb = 5,000 were selected to avoid potential bias from solid linkage disequilibrium. 3) Remove the echo sequence. 4) Remaining SNPs were queried based on GWAS data for platelet traits. 5) The assessment of the strength of each genetic tool was materialized by calculating the F statistic, which was calculated as F = β exposure ^2^/SEexposure ^2^ (Yu et al., 2023). If F > 10, the selected IV has a solid potential for predicting platelet traits. The screening results and F-values are shown in Supplementary Material Excel S1–S6.

## Exposure and outcome

### Statistical analysis

The primary analysis in our study was the inverse-variance weighting (IVW) method, which supposes that all instruments are valid (Burgess et al., 2013). Meanwhile, the weighted median (WM) and MR-Egger methods were used as supplementary analytical methods. According to the assumptions of the IVW method, the estimation of the total effect was realized based on the weighted average of the inverse variance of the instrumental variable effect estimates. IVW has maximum efficacy without horizontal polytomous bias (Burgess et al., 2013). Based on this, IVW can provide the most accurate assessment (Sekula et al., 2016). The WM method studies the bias caused by invalid IVs. If there are 50% invalid IVs, then WM can produce a robust estimate (Bowden et al., 2016). Although the MR-Egger method can study bias due to horizontal pleiotropy (Sanderson, 2021), it is possible that its estimates may not be accurate enough due to the effect of outlier genetic variation (Bowden et al., 2016). The method has the lowest statistical efficacy regarding detection effect (Bowden et al., 2015).

We also have analyzed the sensitivity analysis. Sensitivity analysis is an essential method for assessing potential heterogeneity and horizontal pleiotropy. Cochran’s Q statistic is a widely used tool for evaluating heterogeneity between studies in a meta-analysis (COCHRAN, 1950). This tool can also be used in MR analysis (Greco et al., 2015; Bowden et al., 2019). Meanwhile, leave-one-out analyses were performed to calculate the effect of eliminating one of the individual SNPs on the overall results. All statistical analyses were performed using RStudio version 4.3.1 with the R packages TwoSampleMR version 0.5.6 and Mendelian Randomization version 0.6.0.

### Mendelian randomization analysis process

First, SNPs were selected separately in the exposure selection for genes of different physical activity types, and the six sets of SNPs were picked, as shown in Table 1. The above-selected SNPs were harmonized with effector alleles from the platelet profile database (platelet count, platelet crit, MPV, and PDW), and six sets of genetic tools were selected to elucidate the genetic causality between PA and platelet profiles. Second, when horizontal diversity was present (*p* < 0.05) if it was significant for moderate horizontal diversity, then some SNPs were excluded from the outlier test process (*p* < 0.05) and reanalyzed. If heterogeneity was still substantial after reanalysis, all outliers were excluded. Third, the leave-one-out method was used to analyze whether SNPs still existed, which affected the stability of the results. A final and reliable conclusion could be drawn if SNPs that affected the results were not detected. This study was conducted according to the guidelines of the STROBE-MR statement.

## Results

**FIGURE 2:**
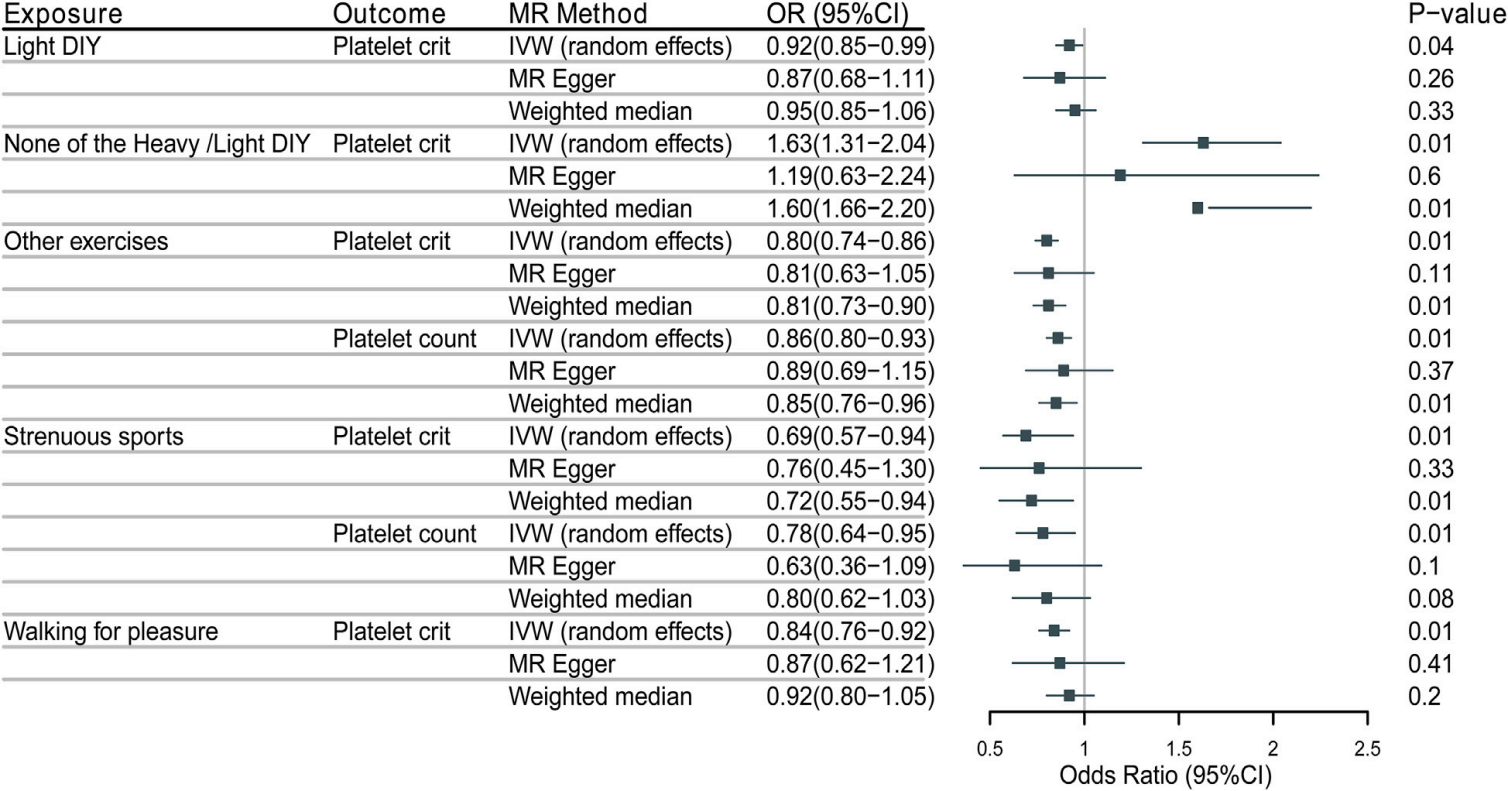
MR Results and positive results of physical activity and platelet traits.

As shown in Figure 2, IVW’s analysis showed that for the types of physical activity in the past 4 weeks, Light DIY (e.g., pruning, watering the lawn) [odds ratio (OR) = 0.9200,5% confidence interval (CI):0.8489-0.9971, *p* = 0.0421] was associated with a reduction in platelet crit in platelet profile.

Neither heavy DIY (e.g., weeding, lawn mowing, carpentry, digging) nor light DIY [OR = 1.6349, 95% CI: 1.3085-2.0427, *p* = 0.0000] was associated with increased platelet crit in the platelet profile. This result was the same as the analysis of WM (OR = 1.6002, 95% CI: 1.6562-2.1970, *p* = 0.0032).

Other exercises (e.g., swimming, cycling, keeping fit, bowling) (OR = 0.8555,95%CI:0.7898-0.9267, *p* = 0.0001) were associated with a decrease in platelet count and the same analyses were obtained with WM (OR = 0.8545,95% CI: 0.7641–0.855). And (OR = 0.7984,95%CI: 0.7384-0.8633, *p* = 0.0000) was also associated with a decrease in platelet crit, with the same results shown by WM (OR = 0.8093,95%CI:0.7294-0.8908, *p* = 0.0001).

Strenuous sports (OR = 0.7814,95%CI:0.6412-0.9521, *p* = 0.0144) were associated with decreased platelet count. Associated also with reduced platelet crit (OR = 0.6870,95CI:0.5691-0.8295, *p* = 0.0000), as analyzed by WM (OR = 0.7171,95%CI:0.5484-0.9377, *p* = 0.0121).

Walking for recreation (not as a means of transportation) was associated with decreased platelet crit (OR = 0.8363,95%CI:0.7599-0.9205, *p* = 0.0003).

**FIGURE 3:**
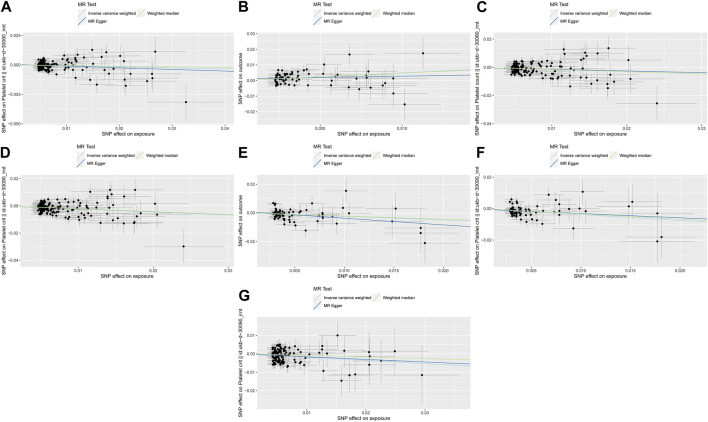
The three lines represent the estimated effect size. Light DIY and Platelet crit **(A)**; None of the Heavy /Light DIY and Platelet crit **(B)**; Other exercises and Platelet count **(C)** and Platelet crit **(D)**; Strenuous sports and Platelet count **(E)** and Platelet crit **(F)**; Walking for pleasure and Platelet crit **(G)**.

**FIGURE 4:**
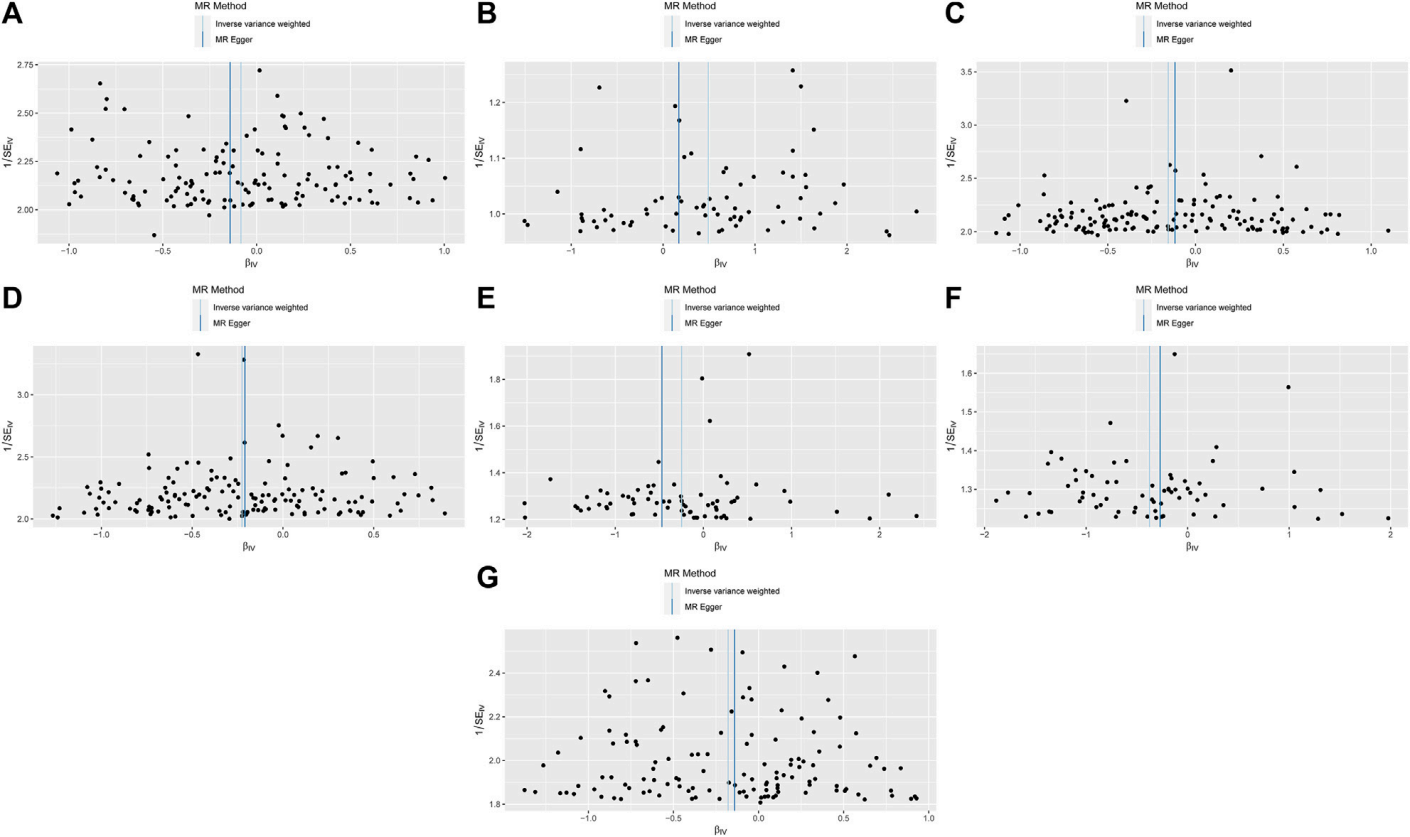
MR funnel-plot.Light DIY and Platelet crit **(A)**; None of the Heavy /Light DIY and Platelet crit **(B)**; Other exercises and Platelet count **(C)** and Platelet crit **(D)**; Strenuous sports and Platelet count **(E)** and Platelet crit **(F)**; Walking for pleasure and Platelet crit **(G)**.

The weighted median method for some of the results needs to be consistent with the primary analysis of the IVW method. A null hypothesis for half of the IVs weakens statistical efficacy. It can result in weak statistical effects and potentially false negatives. As another estimation method, the MR-Egger method may have led to poorer precision of the results because of its wider implementation interval. Moreover, the effect of outlier genetic variation has likely led to the estimation efficacy inconsistent with the IVW method. Based on these two points, the MR-Egger method was mainly used to assess horizontal pleiotropy during the study (The results of heterogeneity detection are shown in Figures 3, 4).

**FIGURE 5:**
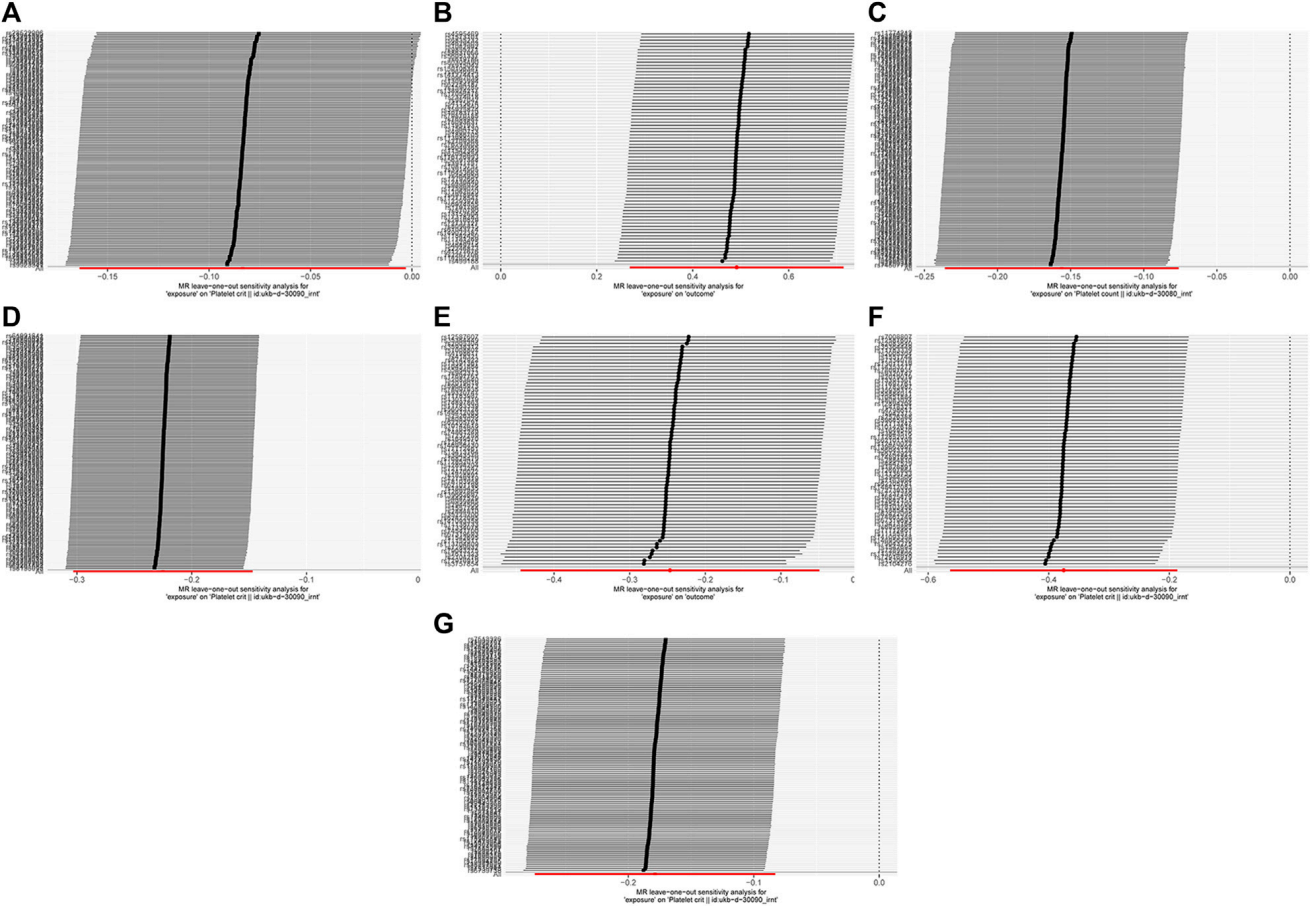
MR leave-one-out sensitivity analysis. Light DIY and Platelet crit **(A)**; None of the Heavy /Light DIY and Platelet crit **(B)**; Other exercises and Platelet count **(C)** and Platelet crit **(D)**; Strenuous sports and Platelet count **(E)** and Platelet crit **(F)**; Walking for pleasure and Platelet crit **(G)**.

It eliminated the outliers that made the results unstable due to anomalies until a stable result was obtained. The sensitivity test results are shown in Figure 5. After reanalysis, based on the results of the heterogeneity and pleiotropy tests, as shown in Table 2, it is unlikely that there is heterogeneity and horizontal pleiotropy (*p* > 0.05). It also showed that the study’s results were almost unlikely to be affected by the effects of heterogeneity bias. The analysis of reverse MR showed no indication of an inverse relationship.

**TABLE 2:** Causal relationship and heterogeneity detection between physical activity and platelet traits.

Exposure	Outcome	nSNPs	Method	OR	LOW	UP	*p*-value	Heterogeneity test (*p*-value)	Pleiotropy test (*p*-value)
Light DIY	Platelet crit	141	MR-Egger	0.8683	0.6793	1.1099	0.2616	0.1249	0.6258
			Weighted-Median	0.9466	0.8461	1.0591	0.3307		
			Inverse variance weighted	0.9200	0.8489	0.9971	0.0421	0.1341	
None of the Heavy/Light DIY	Platelet crit	73	MR-Egger	1.1872	0.6280	2.2444	0.5990	0.5762	0.2968
			Weighted-Median	1.6002	1.6562	2.1970	0.0032		
			Inverse variance weighted	1.6349	1.3085	2.0427	0.0000	0.5722	
Other exercises	Platelet count	142	MR-Egger	0.8896	0.6877	1.1507	0.3745	0.1555	0.7551
			Weighted-Median	0.8545	0.7641	0.9554	0.0058		
			Inverse variance weighted	0.8555	0.7898	0.9267	0.0001	0.1684	
	Platelet crit	142	MR-Egger	0.8112	0.6282	1.0476	0.1110	0.0819	0.8984
			Weighted-Median	0.8093	0.7294	0.8980	0.0001		
			Inverse variance weighted	0.7984	0.7384	0.8633	0.0000	0.0907	
Strenuous sports	Platelet count	70	MR-Egger	0.6254	0.3597	1.0874	0.1009	0.1215	0.4012
			Weighted-Median	0.8010	0.6210	1.0330	0.0770		
			Inverse variance weighted	0.7814	0.6412	0.9521	0.0144	0.1252	
	Platelet crit	69	MR-Egger	0.7628	0.4465	1.3029	0.3251	0.2794	0.6840
			Weighted-Median	0.7171	0.5484	0.9377	0.0121		
			Inverse variance weighted	0.6871	0.5691	0.8295	0.0000	0.3035	
Walking for pleasure	Platelet crit	125	MR-Egger	0.8693	0.6219	1.2124	0.4087	0.0545	0.8185
			Weighted-Median	0.9170	0.8030	1.0471	0.1959		
			Inverse variance weighted	0.8363	0.7599	0.9205	0.0003	0.0613	

## Discussions

This study showed that light DIY, other exercises, strenuous sports, and walking for pleasure were associated with decreased platelet crit (*p* < 0.05) in different physical activities in the past 4 weeks. Neither heavy nor light DIY was associated with an increase in Platelet crit (*p* < 0.05). Other exercises and strenuous sports were associated with decreased Platelet count (*p* < 0.05).

The other exercises and strenuous sports in our study were associated with a decrease in platelet count. A decrease in platelet count is generally defined as a decrease in the number of platelets per unit volume of blood. It was indicated in previous studies that acute exercise leads to a transient increase in platelet count. The mechanism of this increase may be due to the concentration of blood concentration and the release of platelets from the liver, lungs, and spleen (Chamberlain et al., 1990). However, swimming, cycling, keeping fit, bowling and strenuous sports have a different mode and intensity of exercise than the acute mode of exercise, which may be the result of the appearance of a decrease in platelet count rather than the underlying reasons for the increase. In addition, platelet mass in healthy subjects is kept constant by close adjustment, and MPV is negatively correlated with platelet counts (Wiwanitkit, 2004; Adibi et al., 2007; Margetic, 2012). There is substantial evidence that the major determinant of platelet counts is genetics (Daly, 2011), which means that platelet counts have a high genetic heritability (Buckley et al., 2000; Biino et al., 2011). Meanwhile, other acquisitional factors, such as physical activity, smoking status, and alcohol consumption, have been shown to change the platelet counts (Lippi et al., 2014; Hong et al., 2015).

Another characteristic is light DIY, other exercises, strenuous sports, walking for pleasure and None of the Heavy/Light DIY were respectively associated with a decrease and an increase in platelet crit. Platelet crit refers to the volume of platelets in blood volume occupied by platelets in the blood (Budak et al., 2016) and its results are influenced by both PLT and MPV (Giacomini et al., 2001; Chandrashekar, 2013). Thus, the amount of platelets in the blood is maintained in a state of equilibrium by regeneration and elimination under physiological conditions. The decrease in platelet crit due to physical activity or forms of exercise such as pruning, watering the lawn, swimming, walking for pleasure, etc., may be due to an isolated decrease in platelet count or MPV, or a simultaneous decrease in platelet crit. Neither heavy nor light physical activity implies that moderate physical activity may increase platelet crit. Based on the physiological mechanism of acute exercise-induced platelet count increase, it is hypothesized that appropriate physical activity, which shrinks blood concentration, results in an increase in the product of platelet count and MPV.

The physiologic impact of physical activity on platelet traits is a complex and multifactorial process. On the whole, the physiologic significance of moderate physical activity on platelet traits is to maintain moderate platelet numbers and function and to ensure a normal coagulation response. As a general rule, platelets have a lifespan of approximately 7–10 days (Allan et al., 2023). Physical activity helps stimulate the bone marrow to produce new platelets, which has a positive impact on the availability of sufficient numbers of new platelets to enter the circulatory system at the end of the platelet’s life span. The traits of the newly produced platelets are usually maintained within normal physiologic parameters. And moderate physical activity helps to promote good circulation (Duncker and Bache, 2008; De Ciuceis et al., 2023), which ensures that the newborn platelets can quickly get to where they are needed in the body, which is critical for maintaining proper platelet counts and maintaining coagulation. In addition, moderate physical activity induces the release of anticoagulant substances such as nitric oxide from the body, which helps to maintain the thinning of the blood and reduces the potential for platelet adhesion and aggregation within the blood vessels (Barale et al., 2023). It is essential to note that excessive physical activity may hurt platelet longevity and function, for example, platelet index and MPV, among others, can serve as markers of platelet degranulation/activation (Yetkin, 2008) when the release of platelets is not normal, which may be indicative of platelet overactivation. Transitional activation of platelets is more likely to be cleared or consumed more rapidly in the circulation due to aberrant activation, resulting in a reduced lifespan. Therefore, moderate and sustained physical activity may be more conducive to maintaining good platelet traits and platelet physiology.

## Research strengths and limitations

This is the first MR study to examine the causal relationship between physical activity and platelet shape. The main strengths of this study are as follows: First, we have adopted the MR analysis method, which can effectively avoid the interference of confounding factors and reverse causality. Second, we rigorously screened SNPs to assure the independence of IVs and included tools with more powerful genetic functions. Third, most MR analyses reinforce the requirement for consistent β-direction in MR methods, and such consistency was ensured in our study.

Nevertheless, our study has some limitations. First, our study was conducted on a European group, and no differences between genders were distinguished, so it cannot be immediately generalized to other ethnic groups with different cultural backgrounds. Second, DNA methylation, RNA editing, and inactive transposons remain unavoidable problems for MR analysis.

## Conclusion

Our findings support a causal relationship between partial physical activity type and partial platelet traits. For future studies, if more effective methods can be found to reduce bias, more extensive GWAS pooled data are needed to validate our results using MR analysis again. Furthermore, by conducting relevant studies in different ethnic populations from different cultural backgrounds, implementing dynamic monitoring of platelet traits, and further exploring the physiological mechanism and biological mechanisms by which physical activity affects their changes, it can be better applied to clinical research.

## Data Availability

The original contributions presented in the study are included in the article/Supplementary Material, further inquiries can be directed to the corresponding author.
